# Supplementary figures and images for: The Role of miR-375-3p, miR-210-3p and Let-7e-5p in the Pathological Response of Breast Cancer Patients to Neoadjuvant Therapy
Source: Medicina (Kaunas). 2022 Oct 20;58(10):1494. doi: 10.3390/medicina58101494 (PMC9608077; doi:10.3390/medicina58101494)

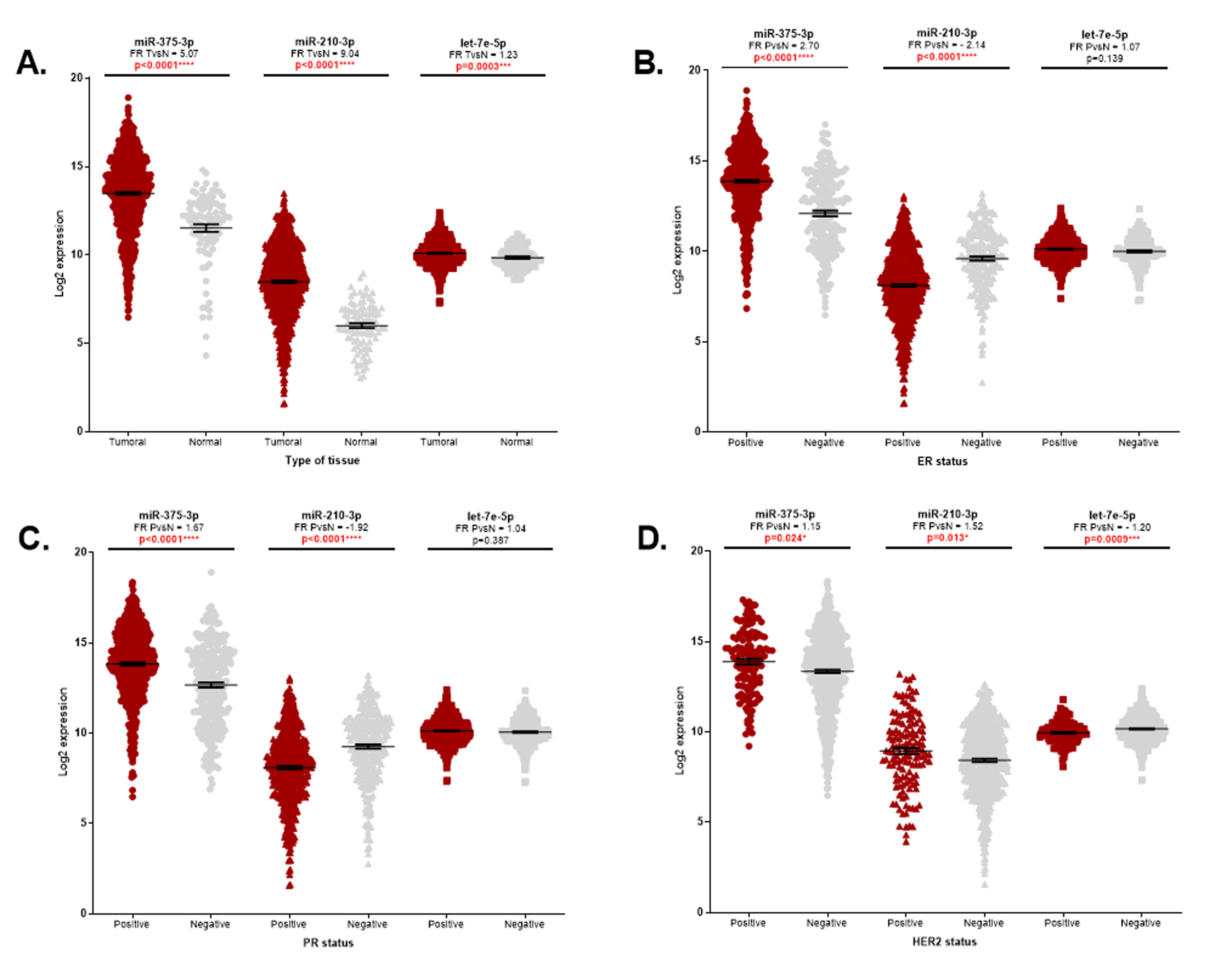

Supplement: Supplementary file 1 [file medicina-58-01494-s001.zip › Supplementary Figure S1.jpg]

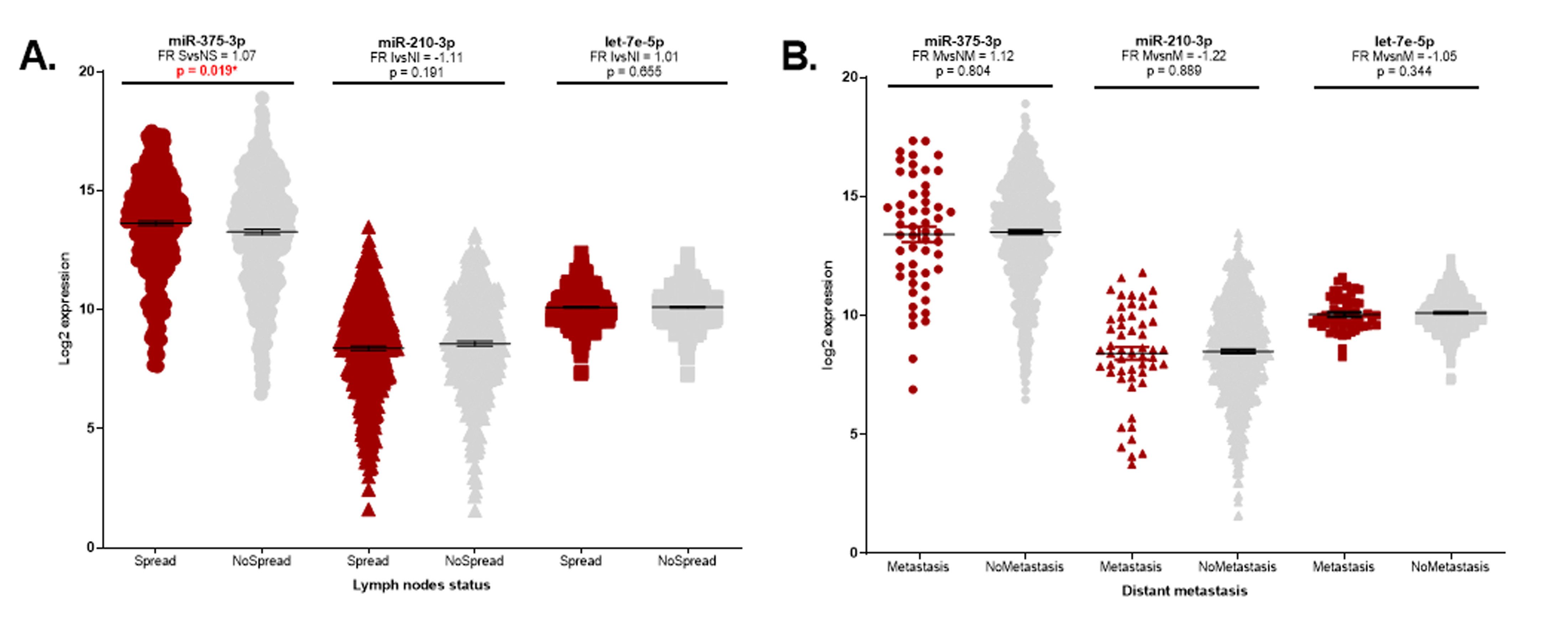

Supplement: Supplementary file 1 [file medicina-58-01494-s001.zip › Supplementary Figure S2.jpg]
